# Nanocellulose Hybrid Lignin Complex Reinforces Cellulose to Form a Strong, Water-Stable Lignin–Cellulose Composite Usable as a Plastic Replacement

**DOI:** 10.3390/nano11123426

**Published:** 2021-12-17

**Authors:** Feitian Bai, Tengteng Dong, Wei Chen, Jinlong Wang, Xusheng Li

**Affiliations:** 1 School of Light Industrial and Food Engineering, Guangxi University, Nanning 530004, China; baifeitian123@163.com (F.B.); dongtengteng6@163.com (T.D.); chenwei1@njfu.edu.cn (W.C.); long05360525@163.com (J.W.); 2 Guangxi Key Laboratory of Clean Pulp & Papermaking and Pollution Control, Nanning 530004, China

**Keywords:** lignin–cellulose composite, bagasse, cellulose, lignin, enzymolysis

## Abstract

The significant challenges in the use of cellulose as a replacement for plastic are its mechanical properties’ degradation and uncontrolled deformation during the rewetting process. Herein, inspired by the reinforcement of cellulose by lignin in natural plant tissue, a strong and water-stable lignin–cellulose composite (LCC) was developed. A nanocellulose hybrid lignin complex (CHLC) created from bagasse residue after enzymatic hydrolysis was added into a pulp of bleached fibre extracted from pine to produce a lignin–cellulose sheet. The lignin as a water-stable reinforcing matrix, via the hydrogen bonding of the nanocellulose in the CHLC with the fibre was efficiently introduced onto the fibres and the fibre network voids. Compared with a typical lignin-free cellulose sheet, the dry strength and wet strength of the LCC were 218% and 2233% higher, respectively. The developed LCC is an eco-friendly and biodegradable alternative to plastic.

## 1. Introduction

Plastics have the advantage of being light and cheap, but they are not easily degraded by microorganisms [1]. Discarded plastics are gradually broken down into microplastics, which accumulate in the environment and food chain, posing a major threat to the ecosystem and human health [2]. Therefore, an eco-friendly and biodegradable replacement for plastics is urgently needed [3]. Cellulose is the most abundant biopolymer on earth, and it is a green and sustainable material that is biodegradable, derivable, renewable, and biocompatible [4,5,6]. It can be produced in large quantities from natural plants through various pulping technologies. With its excellent properties of being low density, having a low thermal expansion coefficient, high strength, high stiffness, and easy deformation, cellulose has the potential to surpass the performance of fossil-based materials in many aspects, showing great potential for replacing plastic [7,8]. However, the use of the cellulose fibre sheet as a replacement for plastic still faces some major challenges, including: (1) the hydrogen bonds between the fibres being broken by water molecules [9], and (2) the inherent structural defect of the fibre network, i.e., the existence of a large number of voids that become water channels, resulting in a degradation in the mechanical properties of the cellulose fibre sheet during rewetting [10].

Enhancing the water-stable mechanical properties of cellulose sheets via both the improvement of the water-stability of bonding between the fibres and the reduction of the inherent voids through physical, chemical, biological, or enzymatic treatments, or a combination thereof, has been extensively studied. The techniques developed include grafting, crosslinking [11], surface coating, ionic liquid treatment [12], mixing coarse and fine fibres [13], and improving the interfibre orientation. However, these techniques often require complex and expensive pretreatment procedures, which reduce the cost-effectiveness and eco-friendliness of cellulose as a plastic replacement for commercial applications. For example, esterification, acetylation [14], atomic deposition, silanization, and polymer grafting can improve the water-stability of cellulose [15], but they reduce the overall mechanical strength of the cellulose sheet. Improving the fibre orientation by wet stretching and shear orientation enhances the strength of the cellulose sheet, but this requires special equipment, thereby limiting large-scale production [16,17]. Ionic crosslinking can effectively improve the water-stability of cellulose sheets [18,19], but not the mechanical strength. Therefore, many challenges remain in the development of a simple and scalable method to enhance the mechanical strength and water-stability of cellulose.

In natural plant tissues, cellulosic fibrils are embedded in a matrix of lignin–carbohydrate complexes, providing excellent water-stable mechanical strength [20,21]. Lignin is a natural adhesive and hydrophobic agent that endows strength, water resistance, and stiffness to plant cell walls [22,23]. Additionally, lignin is the second most abundant biopolymer in biomass after cellulose [24]. When used as a binder, the activated residual lignin in fibrils has been shown to improve significantly the wet strength and water stability of cellulosic materials [25]. This is attributed to the fact that the residual lignin is activated and rich in phenolic hydroxyl during the treatment with nitric acid and hydrogen peroxide, improving the interaction between the lignin and the cellulose [26]. The problem is that the strong interaction between the lignin and the cellulose is broken during the processing from biomass to cellulose [27]. When the cellulose and the lignin are reassembled into cellulosic materials, the strength and water-stability of the cellulosic materials are deteriorated, owing to the poor adhesion between them. This may be due to the incompatible interface properties between more-hydrophobic lignin and less-hydrophobic cellulose, resulting in poor bonding between the fibres [28].

Bagasse is the main solid by-product of the sugarcane industry, with an output of 270 million tons per year in world. Similarly, to other lignocellulosic biomass, the basic components of bagasse are cellulose, hemicellulose and lignin, which can be hydrolyzed by enzymes into fermentable sugars, which can be used as a raw material for the production of bulk products such as ethanol and lactic acid, and has been widely studied. The residue after the enzymatic hydrolysis of bagasse is rich in lignin, and has not been effectively utilized. In order to restore the natural bonding between cellulose and lignin, as found in natural plant tissues, a nanocellulose hybrid lignin complex (CHLC) from the residue of bagasse enzymatic hydrolysis was used as the strengthening matrix of a cellulose sheet. A lignin–cellulose composite (LCC) was manufactured by filtration and hot press treatment. The bagasse was pretreated with phosphoric acid and hydrogen peroxide, and then fibrillated into nanofibrils [29]. The pretreated bagasse was hydrolyzed by cellulase to produce fermentable sugars for bioethanol. The residual nanocellulose and lignin remaining after the enzymatic hydrolysis were mixed together to form a CHLC. The CHLC was used as a reinforcing matrix for a cellulose sheet. The lignin, as an adhesive and hydrophobic agent [30], by virtue of hydrogen bonding between the cellulose in the CHLC and fibres, can easily adsorb onto the fibre surface and fill in the cellulose fibre network. Subsequent hot-press treatment then promoted the softening of the lignin, causing the fibres to be wrapped tightly and evenly by the lignin and ensuring that the cellulose and the lignin were tightly bound together, endowing the LCC with a dense structure. The resulting LCC exhibited excellent physical strength that was far higher than that of many petroleum-based materials. In addition, the hydrophobic lignin endowed the LCC with excellent water-stability, suggesting that it could be used as a plastic replacement to reduce the dependence on fossil resources.

## 2. Materials and Methods

### 2.1. Chemicals and Raw Materials

The bagasse was purchased from Guangxi Guitang Group Co., Ltd. (Guigang, China) and ground by the crusher (800Y-304, Chendu, China); a 40–60 mesh powder was obtained by screening. The cellulose fibre used was bleached pine Kraft pulp obtained from Asia-Pacific Sembo (Shandong) Pulp and Paper Co., Ltd. (Shandong, China). The analytical pure reagents—phosphoric acid (85% *w*/*v*), anhydrous ethanol (98% *w*/*v*), and hydrogen peroxide (30% *w*/*v*)—were purchased from Nanning Blue Sky Experimental Equipment Co., Ltd. (Nanning, China). The CTec2 cellulase was purchased from Novozyme (Shanghai, China). The enzyme activities were 106 FPU/mL. The protein content of the CTec2 was determined to be 115 mg/mL by the Bradford method [31].

### 2.2. Preparation of the Lignin–Cellulose Composite

A total of 300 g bagasse powder, 600 mL H_2_O_2_, and 2400 mL H_3_PO_4_ were placed in a round-bottomed flask and pretreated at 30 °C by stirring at 300 rpm for 16 h. This pretreated bagasse was hydrolyzed in a pH 4.8 citrate buffer with 5 FPU/g cellulase at 50 °C for 8 h. The fermentable sugar solution and the residual solid rich in lignin were obtained by centrifugation.

Different proportions of the residue remaining after the enzymatic hydrolysis to bleached pine Kraft pulp (0:100, 18:82, 28:72, 38:62, and 48:52, relative to the total dry weight) were mixed and diluted with water to reach a final concentration of 1%. The mixture was stirred using a Messmer pulp disintegrator (Mavis Engineering Ltd., London, UK) at 3000 rpm for 10 min. The wet LCC was prepared with a basis weight of 80 g/m^2^ in a normalized Rapid-Köthen handsheet former (PTI, Vorchdorf, Austria) according to ISO 5269/2 (2004), and then cured by hot-press treatment at 150 °C under 4 MPa for 0.5 h to obtain the LCC.

### 2.3. Characterization

The glucose concentration was measured using high-performance liquid chromatography with an HPX-87H column (Agilent, CA, USA) [32]. The cellulase activity was determined by the filter paper method, as described in US NREL28 [33]. The chemical composition of the bagasse and pretreated bagasse was determined using the standard method of the US National Renewable Energy Laboratory [34]. The density was calculated based on the ratio of the grammage to the thickness. The grammage of the LCC was determined following ISO 536 [35]. The thickness of the LCC was determined using a thickness tester (YTH-4, Hangzhou, China).

An electronic universal material testing machine (Instron 3365, Norwood, MA, USA) [36] was used to test the tensile strength properties of the sheets. All of the samples were kept at a constant temperature (25 ± 5 °C) and moisture content (65 ± 10%) for 48 h. In the tensile test, the measured dimensions of the sheets were 20 mm × 5 mm, and the sample was pulled lengthwise at 2 mm/min. A 100 mm × 5 mm sheet was completely immersed in water for 10 min, and then the wet tensile strength was measured as described above. The results are reported as the average of three measurements.

An automatic mercury porosity analyser (Quantachome Poremaster, Canta, FL, USA) [37] was used to determine the pore size distribution. The sheets were cut into 1 cm × 3 cm samples for the analysis.

The contact angle testing [38] was performed using a DSA100 drop shape analyser (Krüss Scientific, Hamburg, Germany). The samples were placed on a glass slide, and 20 µL pure water was carefully injected onto the superior surface of the sample. The contact angle was recorded over time (0–30 min).

In order to evaluate the swelling ratio and water absorption, samples of the sheets were placed in water, and the changes in their thickness and mass were measured over time. The water absorption [39] and swelling ratio [40] of the samples were calculated according to the following formulae:

Water absorption ratio (%) = ((m_2_ − m_1_)/m_1_) × 100 (1)

where m_1_ and m_2_ are the masses before and after immersion, respectively, and

Swelling ratio (%) = ((L_2_ − L_1_)/L_1_) × 100 (2)

where L_1_ and L_2_ are the thicknesses before and after immersion, respectively.

In order to investigate the natural degradation of the materials, straws made of LCC and the commercial straws made of polypropylene (FANJI, Chongqing, China) were buried in the soil of Guangxi University and kept for 42 and 84 days. Their morphology was measured and observed, respectively.

The topography of the sheets was examined with a scanning electron microscope (SEM, SU8220, Hitachi, Tokyo, Japan) [41].

The FTIR spectra (Bruker Technology Vector 33, Ettlingen, Germany) [42] of the bagasse, the pretreated bagasse, and the sheets were obtained using a scan range of 400–4000 cm^−1^ and a resolution of 4 cm^−1^.

The supramolecular structure of the sheets was characterized using an X-ray diffractometer (XRD, MINFLEX 600, Tokyo, Japan) at a scanning speed of 10° min^−1^. The crystallinity indexes (CrI) of the samples were calculated from the XRD patterns according to a conventional method, and the equation as follows [43,44].

CrI (%) = (I_200_ − I_am_)/I_200_ (3)

where I_200_ represents the maximum intensity of the lattice diffraction peak between 2° and 22.5°, and I_am_ represents the intensity of the scattering of the amorphous components in the sample, which was evaluated as the lowest intensity at a 2 Ɵ of 18°.

Samples of the sheets were analysed for their thermal stability in a nitrogen atmosphere from 30 to 700 °C at a rate of 10 °C min^−1^ using a thermogravimetric analyser [45] (TGA, NETZSCH STA 449F5, Selb, Germany).

The Raman spectra [46] were obtained using a laser Raman spectrometer (inVia Reflex, Renishaw, UK) equipped with a confocal microscope (Leica)with a 50× objective lens and a CCD detector. The streamline acquisition mode was used to collect the spectra with a nominal resolution of 4 cm^−1^ in the range of 723 to 1817 cm^−1^. The Raman spectra of the enzymatic residue and the sheets were obtained using an excitation wavelength of 532 nm via an AlGaAs diode laser (Renishaw, London, UK). Raman spectra comprising 2500 points were obtained by scanning a 30 μm × 30 μm area with a step size of 0.6 μm, using 1098 and 1600 cm^−1^ as the characteristic peaks of the cellulose and lignin, respectively.

## 3. Results and Discussion

**Figure 1:**
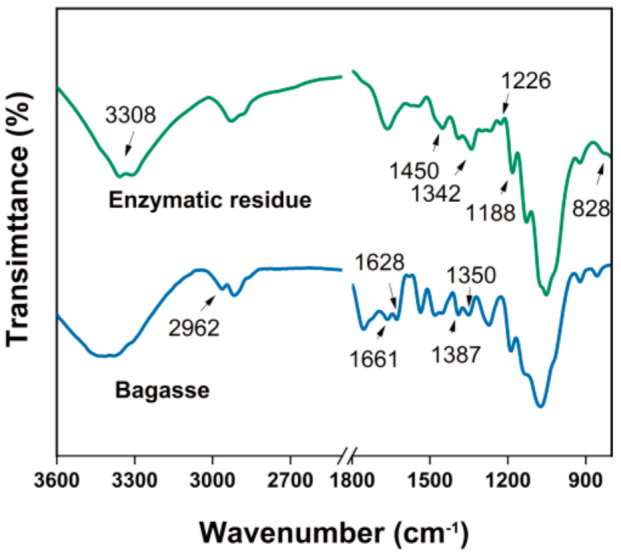
Fourier transform infrared (FTIR) spectra of the bagasse and its residue after enzymatic hydrolysis.

The residue remaining after the enzymatic hydrolysis of pretreated bagasse was composed of 23% lignin, 34% cellulose, and 12% hemicellulose (Table S1). As seen from the FTIR spectra (Figure 1), this residue has characteristic absorption peaks representing cellulose [47] (at 895 (the glycosidic bond of cellulose), 2892 (the C–H tensile vibration of methyl and methylene), 1160 (the C–O–C asymmetric stretching of cellulose), and 1066 cm^−1^ (C–O, C–C stretching vibrations)), hemicellulose [48] (at 1737 (the C=O stretching of the acetyl and urate groups of hemicellulose or the ester bond of carboxyl groups in lignin to fragrant acid and ferulic acid) and 1247 cm^−1^ (the alkyl ester of the acetyl group in hemicellulose)), and lignin [49] (at 823 (the C–H bending vibration of guaiacyl), 1273 (C-O stretching vibration of guaiacyl), and 1637 cm^−1^ (C=O conjugated stretching)). This indicates that the basic structural framework of the lignin, cellulose, and hemicellulose matrix was still present in the residue, which is consistent with the chemical composition analysis. As shown in the XRD results (Figure S1), the CrI decreased sharply from 58.84% to 34.17% after the enzymolysis, indicating that the ratio of amorphous to crystalline regions in the cellulose increased. This may be because amorphous cellulose was mixed with the lignin, which prevented the cellulase from reaching the cellulose [50].

**Figure 2:**
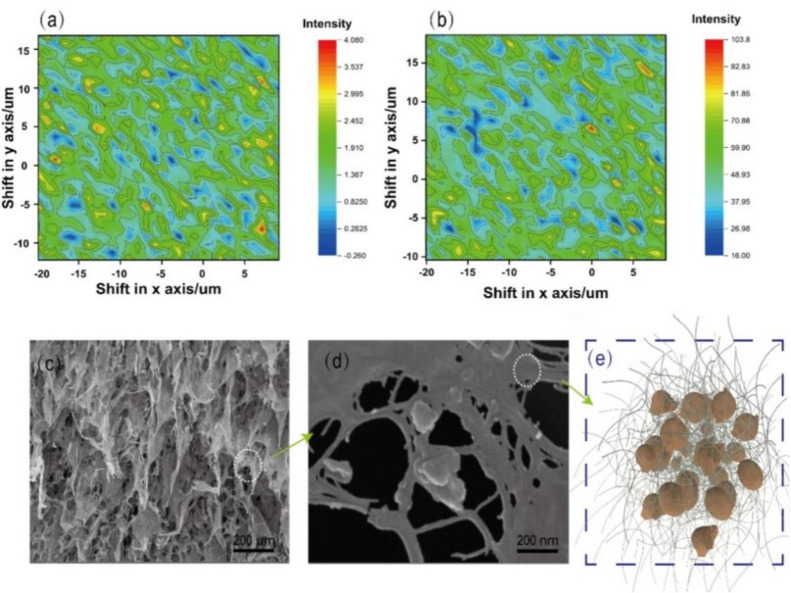
Raman mapping image of (**a**) the cellulose at 1098 cm^−1^, and (**b**) the lignin at 1600 cm^−1^ in the bagasse remaining after enzymatic hydrolysis; (**c**,**d**) SEM images of the residue after enzymatic hydrolysis; (**e**) schematic diagram of the nanocellulose hybrid lignin complex.

Raman spectroscopy (Figure 2a,b) showed that the distribution intensity of the lignin and the cellulose in the residue had full coverage characteristics. This also shows that the cellulose and the lignin in the residue are mixed together. The bagasse pretreated with phosphoric acid and hydrogen peroxide treatment presented a nanofibril skeleton structure (Figure S2).

The morphology of the bagasse changed after the enzymatic hydrolysis, and a lamellar structure was observed in the SEM images (Figure 2c); the nanofibrils’ morphology still exists (Figure 2d). The lamellar structure was attributed to the fact that the nanocellulose self-assembled into films via the strong hydrogen bonding of nanocellulose formed during the drying. The network structure of fibrils in the film is difficult to distinguish, and may be covered by the residual lignin. This indicates that the residual nanocellulose and the residual lignin were mixed together; Figure 2e shows a schematic of the nanocellulose hybrid lignin complex structure.

**Figure 3:**
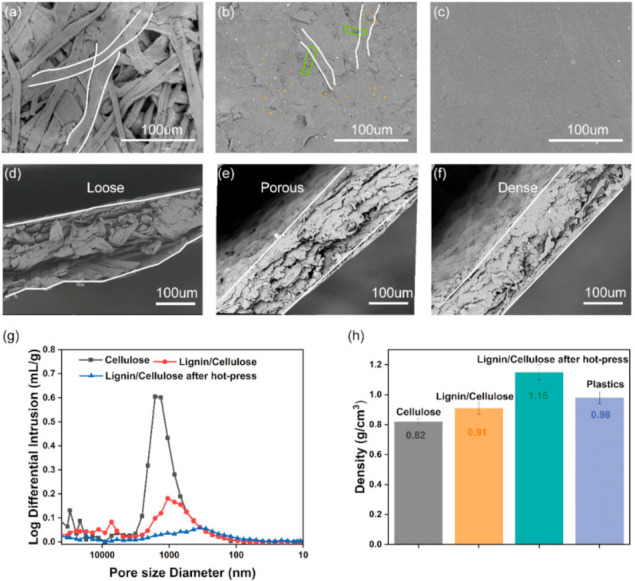
(**a**–**c**) Top view and (**d**–**f**) cross-section of the SEM diagram of the sheets of cellulose, lignin–cellulose, and lignin–cellulose after the hot-press treatment, respectively; (**h**) log differential intrusion image of the sheets; (**g**) density of the sheets.

**Figure 4:**
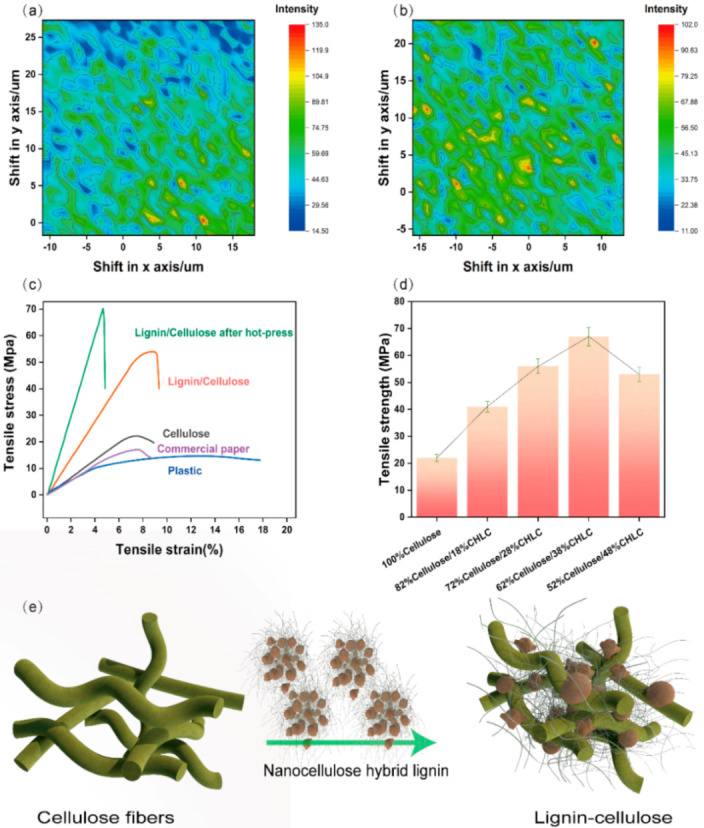
Raman mapping diagram of the lignin–cellulose composite (LCC) (**a**) before and (**b**) after the hot-press treatment; (**c**) tensile strength of the cellulose sheets, plastic (straw), and commercial paper (straw); (**d**) strength of the lignin–cellulose composites with different ratios of the nanocellulose hybrid lignin complex; (**e**) schematic diagram of the nanocellulose hybrid lignin complex-reinforced LCC.

The CHLC and the bleached pine fibre were mixed to form a suspension, and the LCC was formed from the mixed suspension via filtering and hot-press treatment (4 MPa, 150 °C). This process is similar to the traditional papermaking process. As seen in the SEM image (Figure 3a,d), the top view of the initial cellulose sheet has many macroscopically sized fibres that are extruded into a plane and interwoven together to form a sheet with a large number of voids, and the cross section of the cellulose sheet has a loose accumulation of fibres. CHLC was dispersed together with the pine cellulose fibre network and adsorbed onto the fibre surface (Figure 3b,c). Mercury injection experiments showed that the pore size of the LCC before the hot-press treatment (1059.82 nm) was smaller than that of the initial cellulose sheet (1347.52 nm) (Figure 3g). The porosity of the LCC before the hot-press treatment (30.6%) was also smaller than that of the initial cellulose sheet (42.9%) (Table S1). The hot-press treatment transformed the sheet into a dense structure (Figure 2c,f). The porous, rough surface became dense and flat, and randomly distributed fibres were embedded into the CHLC matrix (Figure 3c,f). In the final LCC, the lignin and cellulose were tightly wound and stacked. The pore size was 292.38 nm (Figure 3g), and the porosity was 24.9% (Table S1), resulting in a high density of 1.15 g cm^−3^ for the LCC (Figure 3h). As shown by the Raman spectra (Figure 4a,b), the morphology of the lignin was more evenly distributed from the LCC after the hot-press treatment.

The introduction of the CHLC to enhance the physico-mechanical properties of the cellulose sheet without destroying the cellulose structure is crucial for the construction of high-performance cellulose materials. As the FTIR spectra of the LCC showed (Figure S3), the characteristic absorption peaks at 1408 (CH_2_ symmetric stretching), 1159 (C–O antisymmetric stretching), 1109 (C–OH framework vibration), 1028 (C–O–C pyranose ring vibration), and 872 cm^−1^ (C–H glycoside ring vibration deformation) of cellulose were clearly visible. The characteristic absorption peaks of lignin at 1595 and 1419 cm^−1^ (aromatic ring vibration) and 1265 cm^−1^ (C–O stretching vibration guaiacyl unit), after the addition of CHLC, were also visible. As shown in the XRD image (Figure S4), the variation of the diffraction peaks between the initial cellulose sheet and the LCC corresponding to the (110), (020), and (040) planes of the cellulose I crystal type [51] were unchanged. These data show that the structure of the cellulose was not damaged during the hot-pressing.

The tensile strength of the LCC (70 MPa) is three times of that of a cellulose sheet (23 MPa) (Figure 4c), indicating that the adjacent fibres were strongly bonded, and proving that the CHLC had a reinforcing effect, which was attributed to the fact that the CHLC fills the voids in the fibre network, densifying the LCC during the hot-press treatment (Figure 3). The resulting LCC exhibited excellent physical strength that was far higher than that of many petroleum-based materials (Figure S5). The tensile strength of the LCC was also significantly higher than that of commercial plastic and paper straws (Figure 4c).

Because lignin has an extremely complex structure and dark appearance, the lignin content has an important effect on the optical [52] and mechanical properties of the LCC. The colour of the LCC changes from brown to black with an increase of lignin content (Figure S6). The maximum tensile strength of the LCC also depended on the content of CHLC; the relationship between them appears to be a normal distribution. The tensile strength initially increased with the CHLC content (0–38 wt%), but then decreased significantly with a further increase in the CHLC content (38–48 wt%) (Figure 4d). This trend can be explained by the fact that the tensile strength increases until there is enough CHLC to fill the interfibre gaps, but excessive lignin hinders the bonding between the fibres.

The schematic diagram shown in Figure 3e explains the LCC properties. At first, the CHLC is loaded into the network and surface of the fibre by hydrogen bond interaction between the nanocellulose in the CHLC and the pine cellulose fibres [53]. Then, the lignin particles dispersed in the cellulose matrix undergo a glass transition during the hot-press treatment [54] (Figure S7). The softened lignin adheres to the cellulose network and surface, and the cellulose matrix is tightly bound together [55,56].

**Figure 5:**
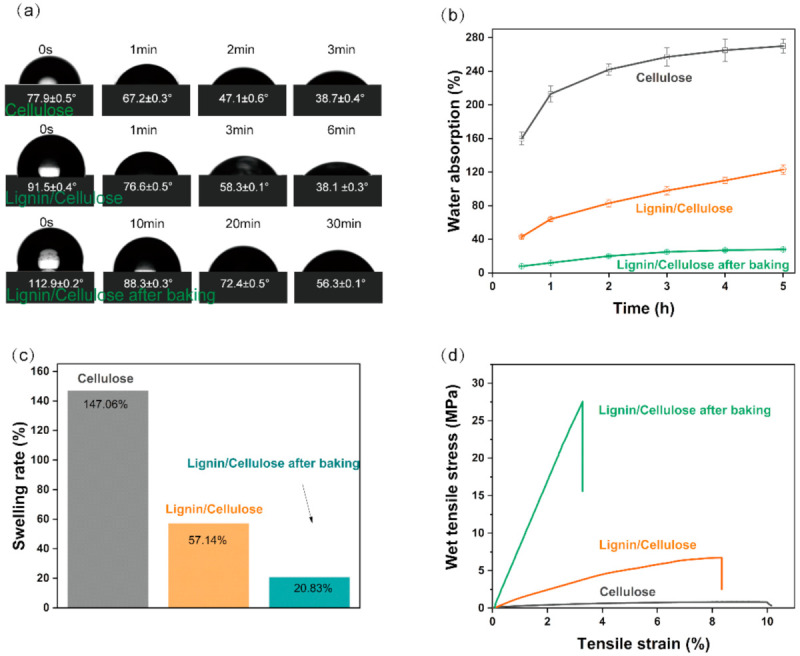
(**a**) Contact angle of the sheets; (**b**) time-dependent change in the water absorption ratios of the sheets; (**c**) thickness-growth percentage of the sheets; (**d**) wet tensile strength of the sheets.

The water-sensitive hydrogen bond is easily broken by water molecules [57] during the rewetting process. Due to the natural moisture resistance of lignin, the LCC can still maintain mechanical strength and water-stability in water to some extent. The initial contact angle of the cellulose sheet was 77.9°, but it gradually decreased to 38.7° after 3 min, owing to the strong hydrophilicity of cellulose (Figure 4b). Similarly, the initial contact angle of the LCC was 112.9°, which remained above 56° after 30 min of moisture absorption, indicating that the LCC had some water resistance. The hygroscopicity test showed that the water absorption of the cellulose sheet was ~260%, while that of the LCC was ~25% (Figure 5b), proving that the lignin is waterproof and thus prevented the fibre from absorbing water. The hygroscopicity tests also showed a swelling ratio of 20.8% for the LCC compared with 174.1% for the cellulose sheet (Figure 5c), demonstrating the reinforcement effect of CHLC to prevent fibre swelling. Compared with the cellulose sheet (1.2 MPa), the LCC exhibited excellent wet strength (~28 MPa), with an increase of 2233% (Figure 5d). Because lignin has a high degree of natural water resistance, it can protect the hydrogen bonds of adjacent fibres in the LCC from being damaged by water molecules, similarly to the reinforcement effect of lignin on cellulose in natural plants [58]. The strengthening mechanism of lignin on LCC water stability may include: (1) lignin, as a more hydrophobic polymer relative to cellulose, acting as a hydroxyl donor unit to endue hydrogen bonds with water resistance [26]; and (2) hydrophobic lignin filling the gaps of the fibre network, and the self-bonding between lignin forming a network to strengthen the fibre network, thus limiting the fibres’ water absorption and swelling [59].

**Figure 6:**
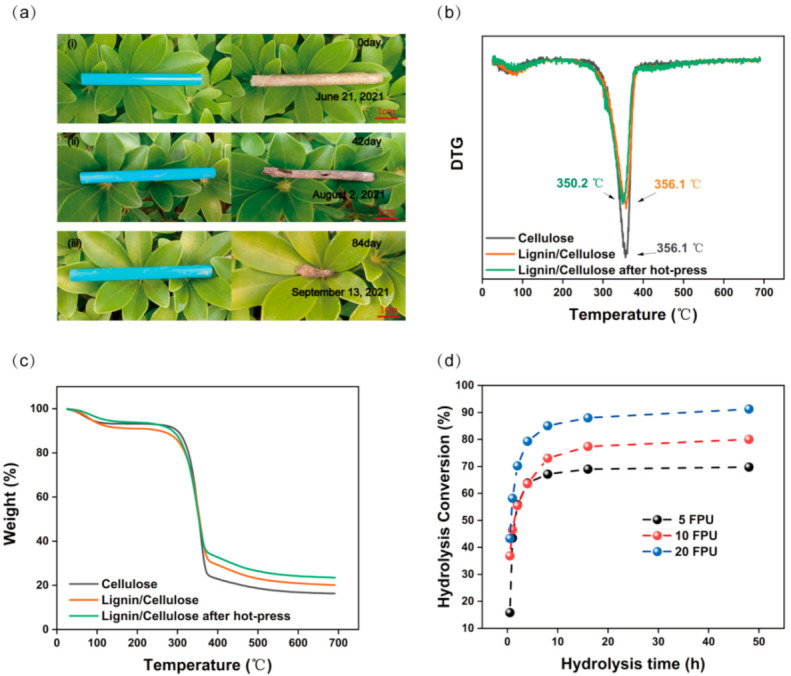
(**a**) (i,ii,iii) The morphology of PP-straw and LCC-straw degraded in nature for 0, 42, and 82 days, respectively; (**b**) thermogravimetric analysis (TGA) and (**c**) differential thermal analysis (DTG) curves of the sheets; (**d**) enzymatic hydrolysis conversion of pretreated bagasse at 5, 10, and 20 FPU/g.

Both the CHLC derived from the residua after the enzymatic hydrolysis of the bagasse and the cellulose fibre extracted from pine are biodegradable biomass, and are nutrients for various organisms (termites, bacteria, etc.) [60]. The LCC is biodegradable in nature (Figure 6a), and harmless to the environment. As shown in Figure 6b, the maximum decomposition temperature of LCC drops slightly after hot-press treatment, which may be due to structural reforming during the hot-press treatment. The maximum decomposition temperature of the LCC was 350.2 °C, which indicated that the LCC had a good thermal stability. The residual mass of the LCC was higher than that of the cellulose sheet (Figure 6c), which was attributed to the higher lignin content in the LCC. The difference of the residual mass of LCC before and after hot-press treatment was attributed to the difference of the initial moisture. The thermal stability of the LCC is a feature that cannot be met by conventional plastics [61].

The strength and excellent water- and thermal-stability of the LCC makes it a promising alternative to plastic for some applications. Considering the deficiencies of cellulose discussed previously, the use of the LCC as a bioplastic will inspire its wider application to many specialized areas, such as in water. In order to test the feasibility of LCC as an alternative to plastic as a straw, an LCC straw was prepared by rolling a wet LCC sheet into tubes and placing it into oven for drying. The LCC straw had good water stability: the suction performance of the LCC straw did not change during 5 h of immersion lag (Figure S8). Therefore, it is reasonable to infer that the LCC is a promising material for biodegradable straws.

Traditionally, high enzyme loading, long enzymatic hydrolysis, or laborious pretreatment procedures have been required to improve the final conversion ratio of biomass to fermentable sugars and bioplastics, making commercial applications unaffordable. As shown in the glucose conversion curve of the pretreated bagasse over time, the enzymolysis was rapid in the first 8 h, then slower from 8 to 48 h (Figure 6d). In addition, low enzyme doses of 5 FPU/g had a higher bond enzyme efficiency per unit than that of the high enzyme doses of 10 and 20 FPU/g (Figure S9). In addition, reusing the reagents of H_3_PO_4_ pre-treated bagasse does not affect the final enzymatic hydrolysis, which can significantly reduce the cost (Figure S10). In the co-production of fermentable sugars and bioplastics, the biomass residue after enzymatic hydrolysis becomes a new source of materials for the reinforcement of cellulose materials, avoiding some inefficient procedures and becoming a promising option for efficient optimization strategies for biomass refining.

## 4. Conclusions

A sustainable and scalable approach to enhance the water-stable bonding between fibres and to reduce inherent voids in cellulose sheets via strengthening by CHLC was demonstrated. Lignin, as a reinforcement matrix, was introduced into the network and surface of the fibre, which significantly improved the tensile strength and water-stability of the LCC. The tensile strength (70 MPa) and thermal stability (>350 °C) of the LCC was significantly higher than that of many petroleum-based plastics. The wet tensile strength (28 MPa) of the LCC was more than 23 times as large as that of a cellulose sheet (1.2 MPa). This newly developed LCC is very attractive as a base material for everyday consumer products such as straws, or as medical packaging to replace non-biodegradable plastics.

## Data Availability

The data presented in this study are available on request from the corresponding author.

## Supplementary Materials

The following are available online at https://www.mdpi.com/article/10.3390/nano11123426/s1. Table S1: Porosity and average pore diameter of the sheets. Figure S1: X-ray diffraction (XRD) images of the bagasse and its residue after enzymatic hydrolysis. Figure S2: Scanning electron microscope (SEM) diagram of pretreated bagasse with phosphoric acid hydrogen peroxide treatment. Figure S3: Fourier transform infrared (FTIR) spectra of the sheets. Figure S4: X-ray diffraction (XRD) images of the sheets. Figure S5: Comparison of the mechanical strength of the LCC and petroleum-based materials. Figure S6: Colours of the lignin–cellulose composite, with different ratios of nanocellulose hybrid lignin complex. Figure S7: Differential scanning calorimetry (DSC) diagram of the lignin in CHLC. Figure S8: Schematic diagram of straws made from different materials, and a water absorption diagram of an LCC straw and a cellulose straw. Figure S9: Initial hydrolysis efficiency of pretreated bagasse with a loading of 5, 10, or 20 FPU/g cellulase for 0.5 h. Figure S10: Enzymatic hydrolysis conversion at 10 FPU/g for 48 h of bagasse pretreated with five cycles of H_3_PO_4_.
