# Supplementary material for: Nanocellulose Hybrid Lignin Complex Reinforces Cellulose to Form a Strong, Water-Stable Lignin–Cellulose Composite Usable as a Plastic Replacement
Source: Nanomaterials (Basel). 2021 Dec 17;11(12):3426. doi: 10.3390/nano11123426 (PMC8708557; doi:10.3390/nano11123426)
Supplement: Supplementary file 1 [file nanomaterials-11-03426-s001.zip › nanomaterials-1489577-supplementary.pdf]

Supplementary Materials:

Nanocellulose hybrid lignin complex reinforces cellulose to form a strong, water-stable lignin-cellulose composite usable as a plastic replacement

Feitian Bai [1,2], Tengteng Dong [1,2], Wei Chen [1,2], Jinlong Wang [1,2] and Xusheng Li [1,2,*]

[1] School of Light Industrial and Food Engineering, Guangxi University, Nanning, Guangxi, 530004, China;

[2] Guangxi Key Laboratory of Clean Pulp & Papermaking and Pollution Control, Nanning, Guangxi, 530004, China

[*] Correspondence: School of Light Industrial and Food Engineering, Guangxi University, Nanning, Guangxi, 530004, China. E-mail address: lixusheng@gxu.edu.cn; Tel.: +86-0771-3237-301

Table S1. Porosity and average pore diameter of the sheets.

Sheet	Average pore diameter (nm)	Porosity (%)
Cellulose	1347.52	42.9
LCC	1059.82	30.6
LCC after hot press	292.38	24.9

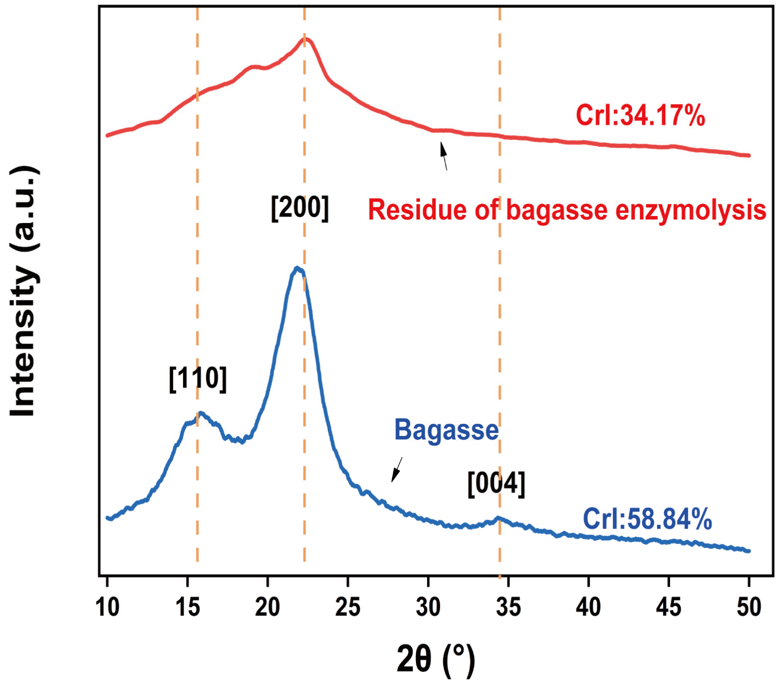

Figure S1. X-ray diffraction (XRD) images of bagasse and its residue after enzymatic hydrolysis.

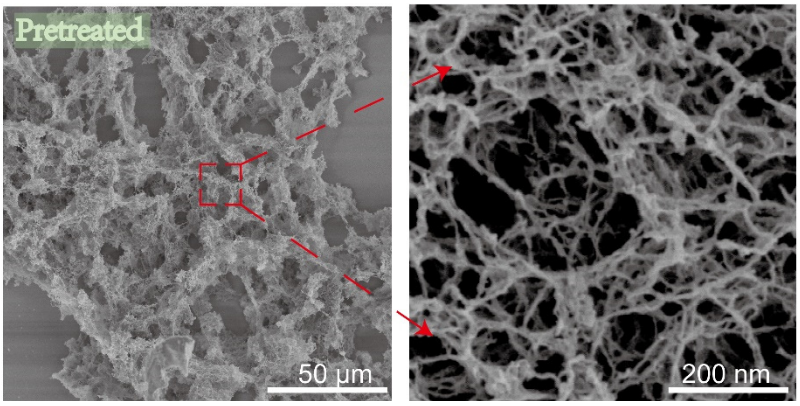

Figure S2. Scanning electron microscope (SEM) diagram of pretreated bagasse with phosphoric acid hydrogen peroxide treatment.

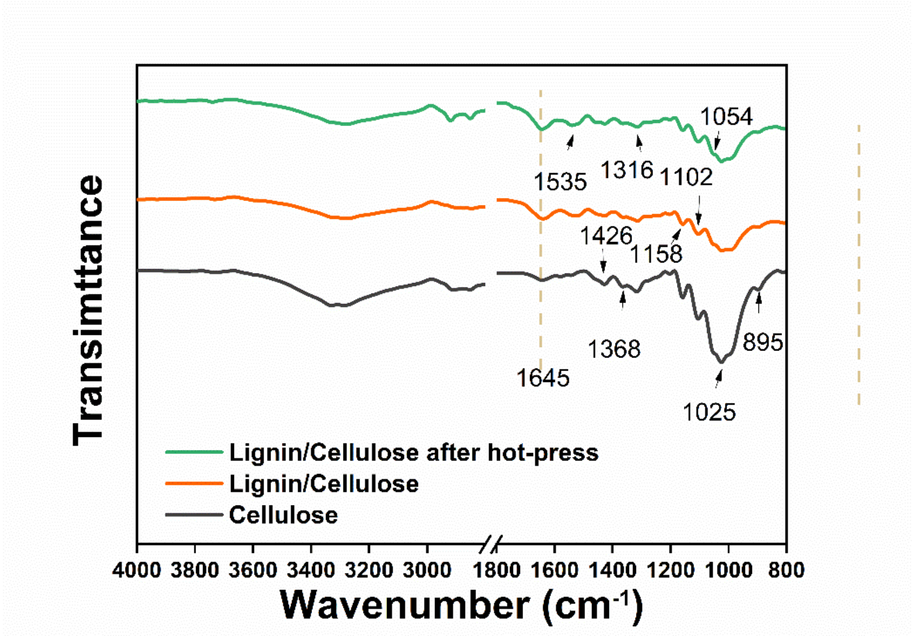

Figure S3. Fourier transform infrared (FTIR) spectra of the sheets.

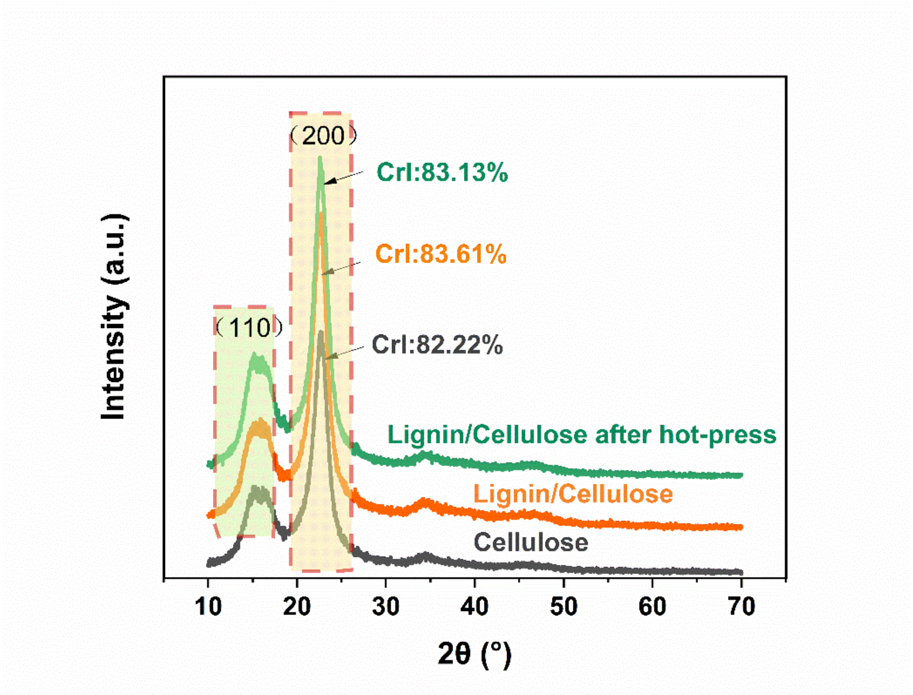

Figure S4. X-ray diffraction (XRD) images of the sheets.

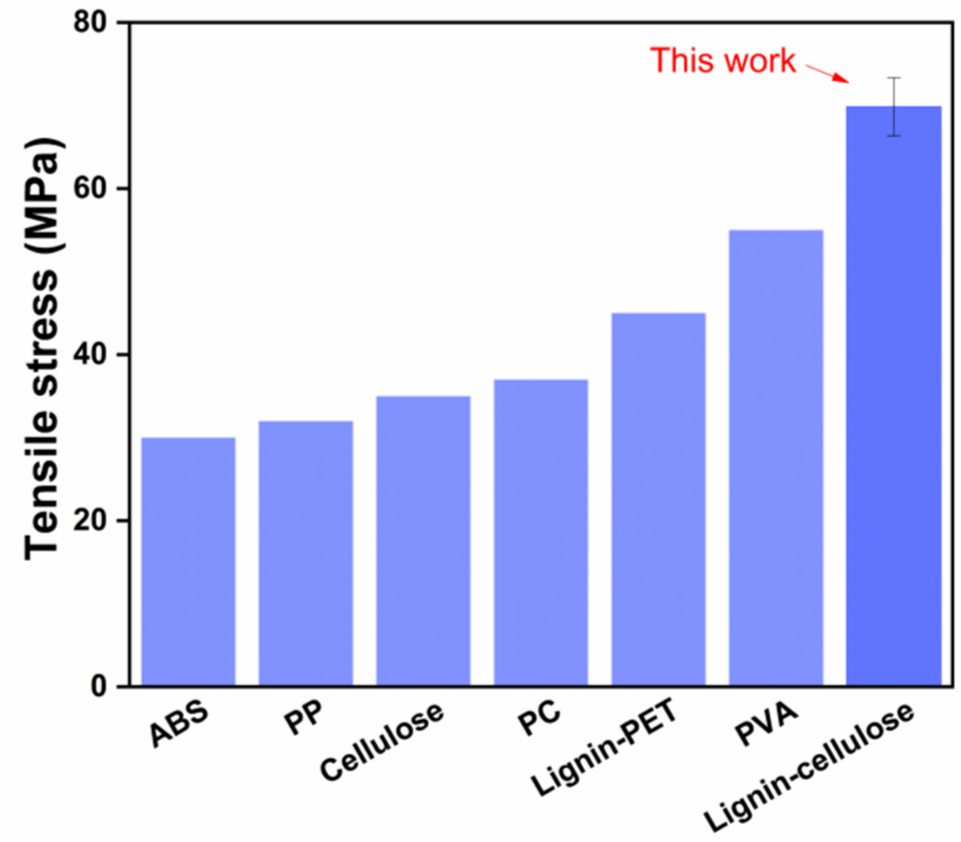

Figure S5. Comparison of the mechanical strength of the LCC and petroleum-based materials

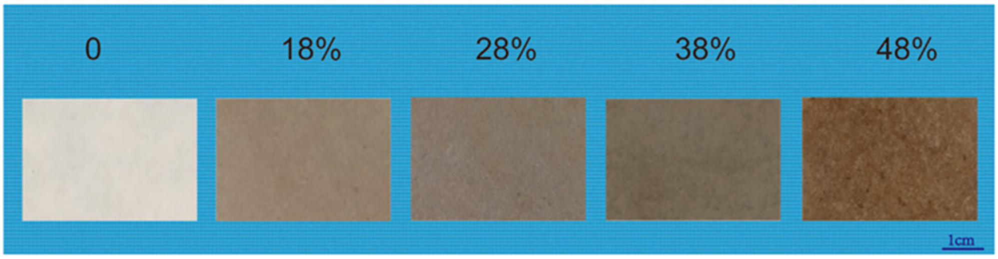

Figure S6 Colours of lignin-cellulose composite having different ratios of nanocellulose hybrid lignin complex.

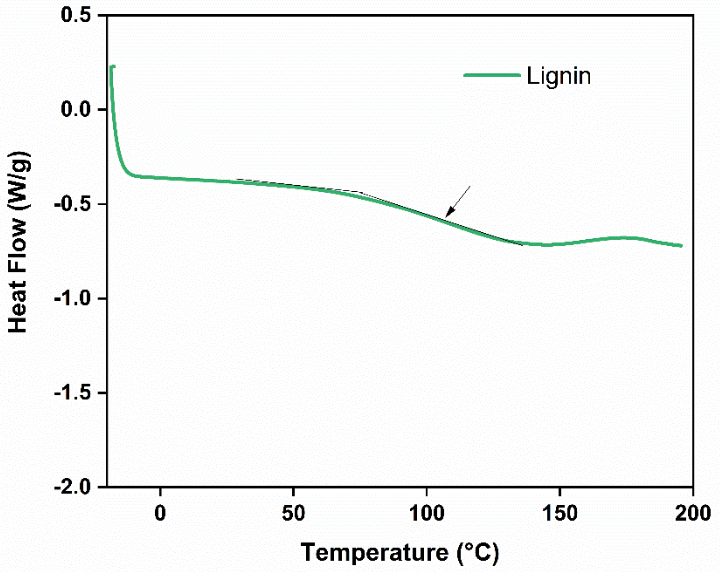

Figure S7. Differential scanning calorimetry (DSC) diagram of the lignin in CHLC

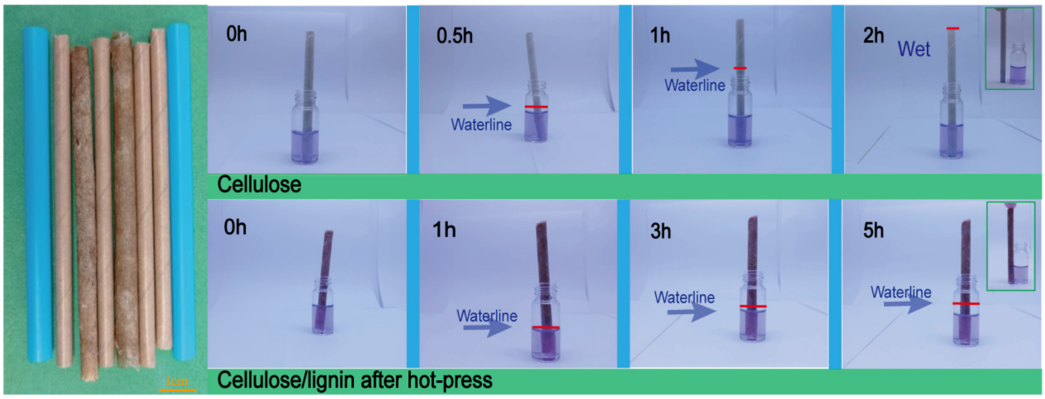

Figure S8. Schematic diagram of straws made from different materials and water absorption diagram of LCC-straw and cellulose-straw.

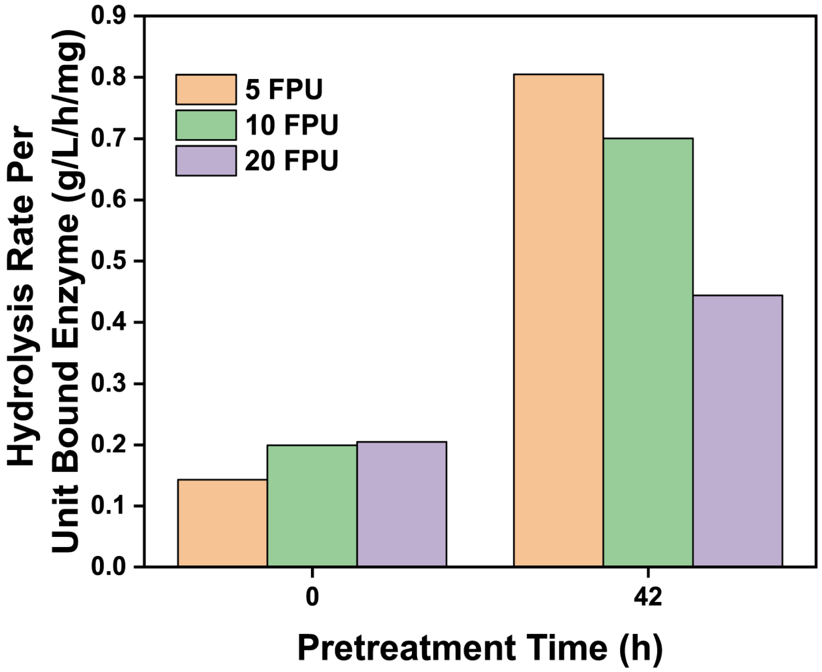

Figure S9. Initial hydrolysis efficiency of pretreated bagasse with a loading of 5, 10, or 20 FPU/g cellulase for 0.5 h.

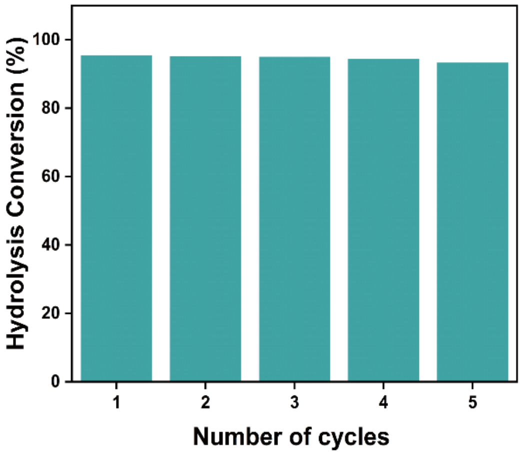

Figure S10. Enzymatic hydrolysis conversion at 10 FPU/g for 48 h of bagasse pretreated with 5 cycles of H_3PO_4.
